# The National Interscholastic Cycling Association (NICA) Mountain Biking Injury Surveillance System (ISS): Analysis of 66,588 Student Athlete-Years of Injury Data

**DOI:** 10.3390/ijerph18115856

**Published:** 2021-05-29

**Authors:** Meredith Ehn, Masaru Teramoto, Daniel M. Cushman, Kristen Saad, Stuart Willick

**Affiliations:** Division of Physical Medicine and Rehabilitation, University of Utah, Salt Lake City, UT 84132, USA; meredith.ehn@hsc.utah.edu (M.E.); masaru.teramoto@hsc.utah.edu (M.T.); dan.cushman.work@gmail.com (D.M.C.); kristen.saad@hsc.utah.edu (K.S.)

**Keywords:** mountain biking, youth mountain biking, injury surveillance system, youth sports, COVID-19, national interscholastic cycling association, sports epidemiology, injury prevention

## Abstract

Interscholastic youth cross-country mountain bike racing in the United States has grown significantly over the past decade, yet little is known about the risk profile in this age group. Aiming to protect participants, we implemented a prospective, longitudinal injury surveillance system for the purpose of better understanding youth mountain biking injuries and implementing safety measures. Data were collected during competition years 2018–2020, totaling 66,588 student athlete-years. Designated reporters from each team received weekly emails with exposure and incident report forms. Variables analyzed included demographic, rider-related, trail-related, and other data. Injury characteristics during the COVID-19 pandemic in 2020 were compared to the years 2018 and 2019. More student athletes participated in the 2020 season (25,261) than in prior seasons (18,575 in 2018 and 22,752 in 2019). During competition year 2020, overall injury proportion was lower (1.7% versus 3.0% in 2018 and 2.7% in 2019). Variables associated with injury, body part injured, type of injury, time-loss, and disposition following injury were similar between all years. Despite the pandemic and resultant changes to competition, student athletes continued to ride their bikes and become injured, but the proportion of injuries differed. This report details injury characteristics in youth mountain bike racing, including a comparison of before and during the pandemic.

## 1. Introduction

Over the past decade, youth cross-country mountain bike racing has become a fast-growing sport in American interscholastic athletic competition [1]. Despite the COVID-19 pandemic, competition year 2020 was no exception. The sport of mountain biking has grown significantly since its modest beginnings in the hills of Marin County, California, over five decades ago [2]. The growth of racing, since the first adult competition in the late 1970s, has followed closely on the heels of the sport’s burgeoning popularity among recreational enthusiasts [2,3]. Youth competition, initially limited by accessibility and therefore participation, lagged behind that of adult competition [4,5].

The growth of competitive youth mountain biking in the United States has trailed behind the growth of the sport in Europe [4]. The National Interscholastic Cycling Association (NICA) was formed in 2009 as the oversight body for middle and high school cross-country mountain bike racing in the United States [6]. As the governing body for interscholastic youth mountain bike competition, NICA is responsible for the administration of all competitive and non-competitive cross-country mountain bike activities, including implementation of rules, league certification, athlete registration, coach registration, and development of training camps and races [6]. In 11 years, NICA has grown from one small league in Northern California to over 30 leagues across the country. By 2020, there were over 25,000 student athletes participating in NICA-sanctioned mountain biking in the United States.

Mountain biking is not without risk. Common injuries include concussion, fracture, dislocation, contusion, and laceration [7,8]. Athletes use specially designed bicycles with shock absorbers and wide, knobby tires to negotiate trails in mountain environments that are often narrow and have natural obstacles, such as rocks, roots, and trees [2,9,10]. The rise in youth mountain bike competition has been accompanied by a concordant rise in mountain biking-related injuries [11,12]. Little is known about the unique injury risk profile encountered in cross-country mountain biking in the adolescent age group. Aiming to protect participants, in 2016, NICA partnered with academic sports epidemiology researchers to design, test, and implement a web-based, prospective, longitudinal injury surveillance system (ISS) to better characterize injuries seen in youth mountain bike racing. After two years of extensive testing and refinement, the ISS went live nationwide in the United States in 2018. The overarching purposes of the NICA ISS are to better characterize and understand injuries in youth mountain biking in order to implement and test data-driven injury reduction strategies. With three full years of data collection now completed, the NICA ISS comprises 66,588 athlete-years of injury data to inform injury reduction strategies, including data collected during the COVID-19 pandemic. This research is imperative in protecting health of student athletes participating in mountain bike, and the NICA ISS is the very first and important step in achieving this goal.

On 11 March 2020, the WHO (World Health Organization) declared the novel severe acute respiratory syndrome coronavirus 2 (SARS-CoV-2 or COVID-19) a global pandemic [13]. Elite and recreational athletes alike were faced with dramatic changes to training and competition that, for many, saw an end to daily habits and life goals that were closely aligned with their sense of self-identity [14,15]. As a result of the COVID-19 pandemic, major world sporting events such as the Tokyo 2020 Summer Olympic and Paralympic Games and 2020 FIFA World Cup were postponed or cancelled [16,17,18]. As competition year 2020 continued on, largely in absence of organized competition for many athletes, the disease proceeded to surge in some areas of the world and abate in others [19,20,21,22,23]. In the United States, states with high rates of disease transmission continued stay-at-home orders through the end of 2020, with schools remaining closed and organized sporting events postponed [24]. Some states were able to safely open schools and return to organized sport competition with appropriate safety measures in place [25]. With varying degrees of school and workplace closures, dramatic changes in organized sports participation, and restrictions on indoor gatherings, the outdoor recreation industry in the United States saw a significant increase in participation during 2020 [26]. Sports amendable to social distancing such as cycling, running, and hiking saw the largest gains in participation [26]. A reflection of this growth in sporting and recreation participation, bicycle sales in the United State rose 75% and 63% in April and June of 2020, respectively, when compared to the same periods in 2019 [27,28]. Children’s bikes were responsible for the largest portion of total sales in June 2020 [28]. It is not surprising then that despite the pandemic, the sport of youth mountain bike racing in the United States continued to grow during competition year 2020 as many Americans made a switch from indoor to outdoor sports that naturally allowed for safer environments for training and racing.

This report briefly describes the NICA ISS with a primary emphasis on the results from the first three years of data collection during competition years 2018, 2019, and 2020, collectively comprising 66,588 student athlete-years. Student athlete-years are defined as one student athlete participating in one year of competition and training [29,30]. Comparison of results obtained during competition years 2018 and 2019 prior to the COVID-19 pandemic, and competition year 2020 largely occurring during the COVID-19 pandemic, are highlighted in the results and discussion. We hypothesized that the NICA ISS successfully collected injury data in 2020 despite the COVID-19 pandemic as well as in 2018 and 2019, and that it allows for the comparisons of injury data across the seasons. To the authors’ knowledge, this is the largest mountain biking ISS in existence. The NICA ISS also tracks injuries among the coaches who ride with the student athletes during practice rides, but presentation of coach injury data is beyond the scope of this manuscript.

## 2. Materials and Methods

The NICA ISS is an electronic ISS designed specifically for mountain biking. The study population included all student athletes formally registered to train and compete in NICA-sanctioned mountain biking practices and races. NICA student athletes are in grades 7–12, approximately ages 12–18 years. Details of the development and implementation of the NICA ISS are described elsewhere [31]. Briefly, it utilizes a web-based survey instrument, REDCap (https://www.project-redcap.org) (accessed on 27 November 2020) [32], to collect data on injuries and exposures in mountain biking during NICA practices and competitions. After extensive testing, the NICA ISS officially began collecting data in January 2018, starting with 23 NICA leagues nationwide. As of Spring 2021, the NICA ISS is used by 29 NICA leagues across the continental United States. The NICA ISS collects injury and exposure data from student athletes as well as coaches. Since an injured rider often sustains multiple injuries in a single injury event (e.g., crash), the NICA ISS specifically incorporated the term “injury event” to collect and quantify injury data. An injury event is defined as “any physical event occurring to a single rider during a NICA-sanctioned practice, race, or other training session that results in physical harm to the participant significant enough to: (1) warrant referral to a medical provider, or (2) lose time from training or competition beyond the day of injury, or (3) miss school or work” [29,31]. For example, a rider may sustain three distinct injuries (e.g., wrist fracture, concussion, and knee laceration) from a single crash. In such cases, one injury event includes three distinct, specific injuries/diagnoses.

Injuries, along with exposures, were reported by a designated reporter on each team. The designated reporter completed an injury report for each injury event via REDCap, using the specific injury report form created by the research team and NICA (Supplementary Material). The designated reporter was a volunteer, often a coach or team manager, trained in sports injury reporting and use of the NICA ISS data reporting form. Multiple injuries were allowed to be reported in a single injury event. Specific injuries (i.e., diagnosis categories) and injured body parts were pre-specified and listed in the injury report form. Along with injury characteristics, the following variables were collected for each injury event: rider demographics, competition division, trail characteristics at the crash location, weather, other factors felt to contribute to the incident (e.g., technical nature of trail, rider inexperience—as reported by the coach), whether an injured student athlete went to an emergency room or not, potential injury mechanisms, and time-loss due to injury (calculated as the days between the date of injury and the date of a student athlete returning to a practice/competition). Additionally, open-ended text fields were required within each injury report, wherein the designated reporter provided written descriptions of an injury event. Aside from the injury-related data above, rider exposures were also reported by the designated reporter on a weekly basis. Exposure was recorded as the number of riders participating in each individual practice ride or race over the week. To promote completeness of the data, we sent each designated reporter a weekly reminder to report any injuries and to complete a weekly exposure report. At the end of each season, all injury events were reviewed in detail, excluding duplicate entries and those that did not meet the injury definition criteria.

Data analysis was performed, mainly using descriptive statistics. Specifically, frequency and proportion/rate were calculated for injury events and specific injuries (injured body parts and diagnoses), along with other injury-related variables mentioned above. Injury event proportion was also calculated as the number of injury events per student athlete-years. As a sub-analysis, these variables were compared between the pre-pandemic seasons (2018 and 2019 combined seasons) and the COVID-19 pandemic year (2020 season) using *χ*^2^ tests. Further, injury characteristics by gender were examined, also using *χ*^2^ tests. Significance tests were performed using Stata 16.1 (StataCorp LLC, College Station, TX, USA). Cleansing and analysis of the text data was performed using R (Version 3.5.1) (R Foundation for Statistical Computing, Vienna, Austria) [33] and its associated packages, including tidytext [34].

## 3. Results

### 3.1. Combined Results 2018–2020

**Figure 1:**
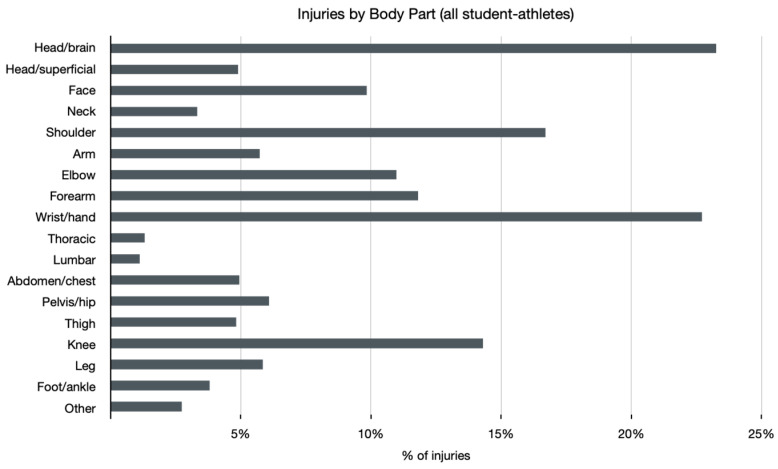
Proportion of student athlete injuries by body part, *n* = 1677 injury events and 2587 injuries. The most commonly injured body parts were head/brain (i.e., concussion or possible concussion, 23.3%), wrist/hand (22.7%), and shoulder (16.7%).

In the first three years of data collection (2018–2020), the NICA ISS recorded 1677 injury events in 66,588 student athlete-years (52,956 or 79.5% males and 13,632 or 20.5% females; Table 1), resulting in an injury event proportion of 2.5%. There was an average of 1.54 unique injuries per injury event (2587 unique injuries in 1677 injury events). An estimated 50% of exposure reports were obtained, resulting in inadequate exposure calculations. Thus, exposure was not included in the analysis. The most commonly injured body parts were head/brain (i.e., concussion or possible concussion, 390 injuries or 23.3%), followed by wrist/hand (381 injuries or 22.7%), and shoulder (280 injuries or 16.7%). Figure 1 summarizes injuries by body part in all student athletes. Contusions and abrasions accounted for 39.7% of all non-concussion injuries. Fractures and dislocations accounted for 26.4% of all non-concussion injuries. Table 2 summarizes injuries by type, excluding concussion or possible concussion.

**Figure 2:**
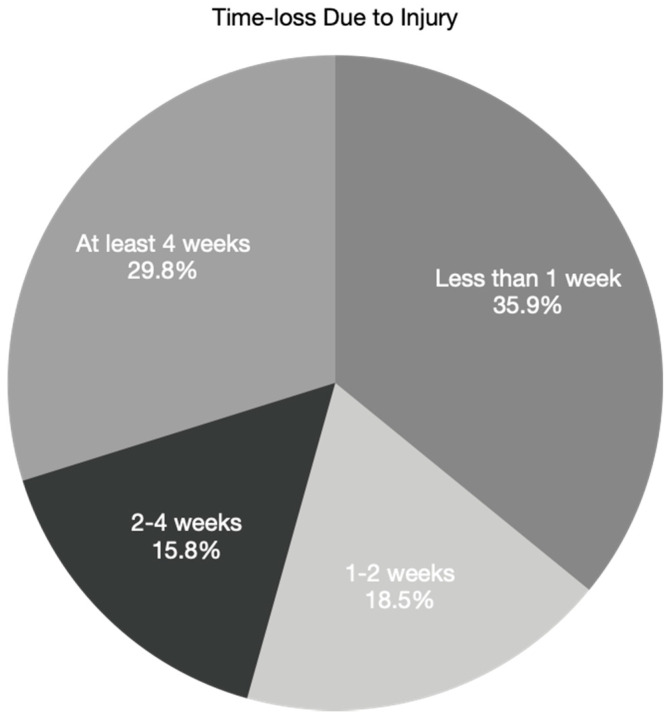
Time-loss due to injury. A total of 35.9% of injury events resulted in time-loss of less than 1 week, while 29.8% resulted in time-loss of at least 4 weeks from riding.

Injured student athletes were unable to complete their practice or race 72.4% of the time (*n* = 1214). Injured student athletes required assisted evacuation from the crash site 13.2% (*n* = 208) of the time, including by ambulance 5.5% (*n* = 93) of the time and by helicopter 0.5% (*n* = 8) of the time. While 49.3% (827 cases) of all injury events resulted in an emergency room visit, only 3.3% of injury events (56 cases) resulted in hospital admission. As seen in Figure 2, 35.9% (492 cases) of injury events resulted in time-loss from riding of less than one week, whereas 29.8% (409 cases) resulted in time-loss of at least 4 weeks. Injuries were season-ending for 15.2% (*n* = 255) of student athletes.

For all years 2018–2020, injury events occurred during a team practice on mountain bike trails 60.2% (1009 events) of the time and during a race 25.0% (419 events) of the time. On the basis of incline, we found that 52.1% (873 cases) of injury events occurred while riding downhill, 31.8% (534 cases) occurred on flat terrain, and 6.4% (108 cases) occurred while riding uphill. With respect to trail familiarity, 74.8% (1254 cases) of injury events occurred on a trail with which the student athlete was familiar. Other common factors reported to be associated with injury events included inexperience of the student athlete (22.5% or 378 cases), technical nature of the trail (19.7% or 331 cases), and negotiating a turn (19.4% or 325 cases), while 27.1% (454 cases) of injury events were not associated with any particular cause.

For all three years combined, there was a small but statistically significant difference in injury event proportion between male and female riders (2.4% vs. 2.9%, respectively; *p* = 0.004). Additionally, some injury patterns in student athletes differed significantly by sex; 22.9% of injuries in female riders were to the lower limb compared to 17.4% in male riders (*p* = 0.003). Male riders experienced a higher proportion of shoulder injuries compared to female riders (18.2% and 11.8%, respectively; *p* = 0.003). Student athletes entered in the freshman, sophomore, and varsity divisions experienced a significantly higher rate of injury (2.8%, 4.1%, and 3.5%, respectively) in comparison to the other divisions (middle school and junior varsity with rates of 2.3% and 2.1%, respectively; *p* < 0.001).

The greatest cumulative mean time-loss occurred in 8th graders (32.55 days, 95% CI 26.54–38.56), and the least cumulative mean time-loss occurred in 12th graders (18.05 days, 95% CI 14.50–21.60). Additional analysis revealed that the highest rate of concussion/presumed concussion injuries occurred in junior varsity class racers (30.7% of JV racers), while the lowest proportion occurred in 7th grade class racers (12.4% of 7th graders).

Analysis of free text entries from combined 2018 and 2019 data showed “front tire washed,” “single track trail,” and “front wheel washed” were the most commonly occurring three-word phrases in the text descriptions used to describe the cause of injury events in student athletes. On the basis of a sub-analysis, we found that loss of control of the front wheel or tire is frequently associated with events leading to injury in both male and female riders. This loss of control, commonly referred to as “washing out,” is the result of loss of traction of the front tire, causing the bike to slide sideways away from the rider when cornering.

### 3.2. Comparison of Combined 2018 and 2019 Results to 2020 Results during COVID-19

**Figure 3:**
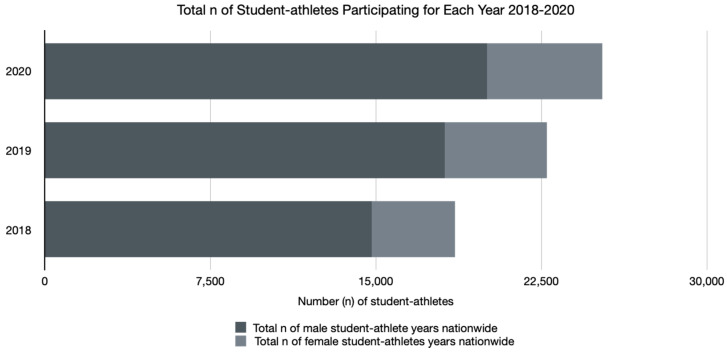
Total number of student athlete-years nationwide for each year (2018–2020).

The total number of registered NICA student athletes rose steadily with each year of data collection, as displayed in Figure 3. The proportion of male and female racers remained consistent with males, comprising 79.7% in combined years 2018 and 2019 and 79.3% in 2020; similarly, females comprised 20.3% of student athletes in combined years 2018 and 2019 and 20.7% in 2020. Table 3 summarizes the total number of student athletes as well as number and proportion of male and female racers during each year (2018–2020).

**Figure 4:**
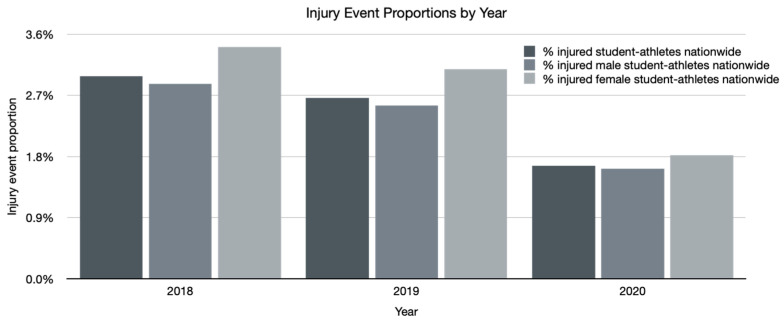
Injury event proportions by year. Injury event proportions decreased for both males and females from 2018 to 2020.

Despite a rise in total number of registered student athletes, there was a decrease in overall injury event proportion between combined years 2018 and 2019 and 2020 (2.8% vs. 2.1%, respectively; *p* < 0.001). Sub-analysis of male and female injury event proportions showed that in combined years 2018 and 2019, 3.2% of female and 2.7% of male student athletes were injured; this proportion dropped to 2.3% in females (*p* = 0.002) and 2.0% in males (*p* < 0.001) during 2020. Figure 4 summarizes injury event proportions by year.

The proportion of unique injuries per injury event did not change between the years (1.5% during combined years 2018 and 2019 vs. 1.6% during 2020; *p* = 0.185). The proportion of injury events that occurred during a team practice on mountain bike trails significantly increased between combined years 2018 and 2019, and 2020 (55.4% vs. 70.7%, respectively; *p* < 0.001), whereas the proportion of injury events that occurred during a race decreased between combined years 2018 and 2019, and 2020 (29.9% vs. 14.2%, respectively; *p* < 0.001). There were 104 high school races nationwide in 2018 and 111 races in 2019; in 2020, there were only 22 high school races nationwide.

## 4. Discussion

This research is very important to better protect student athletes by gaining further understandings of youth mountain biking injuries, through a nationwide, large scale ISS. The successful implementation of the NICA ISS over the past three years has been made possible by the accelerating work of sports epidemiologists worldwide; technology that allows large-scale data collection across a wide geographic distribution; and, most importantly, consistent support for and participation in the ISS by the NICA community. This ISS came about as the result of a strong desire by many within the mountain biking and sports medicine communities to protect the health and safety of young riders. After three years of data collection, this efficient, nationwide ISS for mountain biking has been successfully implemented with early results beginning to inform injury prevention strategies in this promising group of young athletes. Despite changes to racing and training patterns during the COVID-19 pandemic, this ISS was able to continue uninterrupted, providing valuable information about characteristics of riding and subsequent injury risk during the pandemic and affirm some trends identified during the two pre-pandemic years of data collection.

As explained in the Methods section, exposure and injury data were tracked via a web-based injury reporting system with data entered weekly by a designated reporter from each team. For the purpose of this ISS, exposure was defined as one student athlete participating in one practice or race. Unfortunately, during the first 3 years of data collection, compliance with completing exposure reporting was highly variable from team to team (average 50%) and was insufficient to calculate injury incidence. Therefore, only injury proportions and injury event proportions have been reported. The reason for the variability in exposure reporting is under review. Recognizing the importance of capturing exposure data [35,36], NICA is working to increase compliance with exposure reporting. A more detailed discussion of the NICA ISS methods are described elsewhere [31].

### 4.1. 2018–2020 Combined Results

We believe that the quality of injury data for all years (2018–2020) is considered good on the basis of our methodological approaches to data collection implemented in the NICA ISS. The injury definition is aligned with similar systems [36,37] and designed to exclude inconsequential injuries. Designated reporters consistently provided detailed accounts in the open-ended text entry fields. Analysis of the text entry fields has enabled identification of additional common injury characteristics, namely, that of the front wheel washing out. Formal data validation has been delayed due to the COVID-19 pandemic but will be completed in the future.

For all years (2018–2020), overall injury event proportion has continued to be relatively low for student athletes (2.5%). This injury event proportion suggests that on average, 1 out of 40 student athletes per year will have an injury event. Concussion/possible concussion was the leading diagnosis in this cohort of youth mountain bikers. Additional analysis is needed to understand why junior varsity class racers experience the highest rate of concussion/presumed concussion injuries. Future plans include studying factors leading to concussion so that results may be used to inform interventions aimed at decreasing the rate of concussion. Upper limb injuries, specifically to the wrist/hand and shoulder, were more common than lower limb injuries in student athletes. This is similar to reports of mountain biking injuries in other populations [12,38,39,40,41,42].

Injury patterns differed by sex in student athletes, a finding consistent across all three years of data collection. Females sustained more lower limb injuries than males, while males sustained more shoulder injuries than females. Nelson and McKenzie found similar patterns in upper versus lower limb injuries between male and female pediatric recreational mountain bikers treated in the emergency room [39]. Similar upper and lower extremity injury patterns were identified on analysis of the NICA coach injury data (not presented here). Differences in injury patterns may be reflective of the way each rider maintains position on the bike and the manner in which different riders fall. Bike geometry and relative position on the bike determine a rider’s center of mass. This may dictate whether a rider falls forward (sustaining an injury to the upper extremity) or to the side (landing on a leg or foot). Video capture of injury events could help elucidate these differences but the practical application of outfitting many miles or even select sections of mountain bike trail with a camera system is neither financially nor logistically feasible at this time.

The proportion of male versus female student athletes has remained consistent across each of the three years of data collection, with males comprising about 80% of the total number of student athletes and females about 20%. NICA is working to increase the number of female student athlete participants through increasing the number of female coaches, initiation of programs such as Girls Riding Together (GRiT), and other initiatives [43]. GRiT is NICA’s initiative to increase female participation to 33% overall by 2023. GRiT’s goal is to empower female student athletes on and off the bike.

Across all three years of the NICA ISS, many injuries were relatively minor, including abrasions and contusions, leading to less than one week of time-loss. However, some injuries were more significant, including concussions/possible concussions, fractures, and dislocations, and resulted in four or more weeks of time-loss. Additional analysis is needed to understand why 8th graders experienced the greatest mean cumulative time-loss (32.6 days), nearly double that experienced by 12th graders (18.1 days). There were no catastrophic injuries such as spinal cord injury, severe traumatic brain injury, or death reported. Efforts to prevent more severe injuries will be prioritized in the future.

Several factors associated with injury occurrence are being used to inform injury prevention strategies now and in the future. Injuries occurred more often while riding downhill, but many did occur on flat and uphill sections. Rider inexperience and technical nature of the trail have been consistently identified as factors contributing to injury. Washout of the front tire is commonly described in text entry fields as contributing to injury. All of these factors highlight a mismatch between riders’ self-perception of ability versus the skill required for the terrain and speed they are riding as a primary causative factor for crashing. The interactions between rider, bicycle, speed, and trail characteristics have important implications for injury prevention [44] and warrant further study. Injury prevention efforts will emphasize the need for additional skills training for riders with the first prospective, controlled injury prevention intervention scheduled to begin in the fall of 2021.

### 4.2. Comparison of Combined Years 2018 and 2019 to 2020 during COVID-19

In comparison to the two combined pre-pandemic years of 2018 and 2019, competition year 2020 saw an increase in the number of registered riders, possibly due to a continuation of the trend toward increasing student athlete enrollment each year. It is equally possible that this increase in participation was due to the cancellation of and shift away from other organized sporting events or reflective of the nationwide trend that saw many Americans making the switch from indoor to outdoor sports that naturally allowed for safer environments for racing and training with respect to the risk of transmission of the coronavirus [26]. The proportion of male to female student athletes (≈80% vs. ≈20%, respectively) remained constant. There were 1.5 unique injuries per injury event in combined years of 2018 and 2019, compared to 1.6 unique injuries per injury event during 2020, an insignificant difference.

Despite an increase in the total number of participants during the COVID-19 pandemic in 2020, the injury event proportion decreased for both males and females, likely the result of decreased exposure due to cancellation of some practices and races, particularly in the spring and summer of 2020 when lockdowns were most common across the United States [24]. Exposure data would have been especially helpful. However, we can look to both the total number of races conducted as well as proportion of injuries that occurred during racing vs. training to gain more insight. There were nearly five times as many races conducted in each 2018 and 2019 (104 and 111 races, respectively) than in 2020 (22 races). During combined years of 2018 and 2019, injury events occurred during a team practice on a mountain bike trail 55.4% of the time and during a race 29.9% of the time; in comparison, during 2020, injury events occurred during a team practice 71.6% of the time and during a race 12.7% of the time. This is suggestive of less time spent racing during 2020 and more time spent training with the team. Races consist of large gatherings with many teams, with subsequently increased risk of viral transmission, even outdoors. In contrast, it is easier to maintain physical distancing during smaller team rides.

There were no significant between-year differences in the proportions of body parts injured, type of injury (contusion/abrasion/fracture/dislocation), incline at the time of crash, mode of transportation from the crash site, need for emergency room care, or time-loss. The differing pattern of injury characteristics between the sexes was also unchanged between pre-pandemic and pandemic years.

## 5. Limitations

The NICA ISS has several of the same limitations that challenge other, large sports epidemiology investigations in youth athletes. First, data are not always entered by medical personnel, but rather by a volunteer designated reporter on each team who may or may not be medically trained. Unfortunately, it is not practical in a study of this size to obtain formal medical records for all injuries or have a medically trained individual complete the injury report forms for all teams. On the other hand, our method makes it possible to collect and analyze data across the NICA leagues nationwide. Further, all designated reporters receive training on how to complete the injury reporting forms. As mentioned previously, we plan to conduct a formal data validation process, which has been delayed by COVID-19, in the near future. Second, teams have not provided adequate exposure data during the first 3 years of data collection to allow for calculation of injury incidence rate. Therefore, we have only reported injury proportions in this manuscript. NICA is making extensive efforts to increase compliance with exposure reporting from all teams. Third, it cannot be assured that all injuries that meet the inclusion criteria were reported, potentially causing an underestimate in the total number of injuries. However, we believe that under-reporting injuries is not a serious issue, as teams are mandated by NICA to report all qualifying injuries for the purposes of insurance reporting as well as for the ISS, and frequent reminders are provided to coaches, designated reporters, parents, and student athletes about complying with this mandate. Lastly, the NICA ISS focuses on acute traumatic injuries, which by far account for the majority of injuries in this sport. The inclusion of overuse injuries and medical illness remains under consideration for the future.

## 6. Conclusions

A prospective, nationwide injury surveillance system for organized youth mountain biking was successfully implemented in the United States. During the first three years of data collection, 1677 injury events resulted in 2587 unique injuries during 66,588 student athlete-years of participation. The overall injury proportion was relatively low, with 2.5% of student athletes per year sustaining an injury event. Each injury event (or crash) resulted in an average of 1.54 unique injuries. While many injuries were relatively minor (contusions and abrasions), resulting in a short period of time-loss from riding, more severe injuries such as concussion, fractures, and dislocations accounted for half of all injuries. Nearly half of all injuries resulted in a visit to an emergency room, and nearly 30% resulted in at least 4 weeks of time-loss from riding. While the overall injury proportions of female and male student athletes were similar, injury characteristics differed between the sexes, with female riders sustaining more lower limb injuries and male riders sustaining more upper limb injuries. Finally, the pandemic was associated with a lower injury event proportion during 2020 compared to the first two years of data collection in 2018 and 2019, likely as a result of decreased exposure due to cancellation of some practices and races, particularly in the spring and summer of 2020. These data will inform future injury reduction interventions and serve as baseline data against which to compare injury data following such interventions. The first prospective, controlled injury prevention intervention was postponed due to the pandemic and is now planned for implementation in the autumn of 2021.

## Figures and Tables

**Table 1:** Student athlete characteristics. Total *n* and percentage of student athletes enrolled in the NICA ISS during three years of data collection, 2018–2020.

Student-Athlete Characteristics 2018–2020		
Characteristic	*n*	%
Sex		
Male	52,956	79.5
Female	13,632	20.5
Division		
Middle school	26,300	39.5
Freshman	13,016	19.5
Sophomore	5079	7.6
Junior varsity	19,279	29.0
Varsity	2479	3.7
Other/not reported	435	0.7

**Table 2:** Student athlete injuries by type, excluding concussion, *n* = 2108 injuries. Contusions and abrasions accounted for 39.7% of all non-concussion injuries in males and females collectively, while fractures and dislocations accounted for 26.4% of all non-concussion injuries. Total percentage exceeded 100% as multiple individual injuries can occur in a single injury event.

Injuries by Type (Excluding Concussion)						
	All Student-Athletes		Male Student-Athletes		Female Student-Athletes	
	*n*	%	*n*	%	*n*	%
Contusion	360	21.5%	264	20.5%	96	24.6%
Abrasion	476	28.4%	381	29.6%	95	24.4%
Laceration	267	15.9%	205	15.9%	62	15.9%
Ligament sprain	169	10.1%	126	9.8%	43	11.0%
Muscle/tendon strain	81	4.8%	64	5.0%	17	4.4%
Fracture	505	30.1%	415	32.2%	90	23.1%
Dislocation	51	3.0%	41	3.2%	10	2.6%
Unknown	106	6.3%	76	5.9%	30	7.7%
Other	93	5.5%	65	5.1%	28	7.2%
Total and % of injury events	2108	125.7%	1637	127.2%	471	120.8%

**Table 3:** 2018–2020 student athlete-years, showing a consistent proportion of male and female student athletes.

Total n of Student-Athletes Nationwide 2018–2020						
	2018		2019		2020	
	*n*	%	*n*	%	*n*	%
Total *n* of student-athlete years nationwide	18,576		22,752		25,261	
Total *n* of male student-athlete years nationwide	14,819	79.77%	18,108	79.59%	20,030	79.3%
Total *n* of female student-athletes years nationwide	3757	20.23%	4644	20.41%	5231	20.71%

## Data Availability

Data are not available to the public due to ethical, legal, and privacy issues associated with protected medical information of study participants.

## Supplementary Materials

The following are available online at https://www.mdpi.com/article/10.3390/ijerph18115856/s1, File S1: NICA Safety Reporting Incident/Exposure Report Form.
