# Supplementary material for: The National Interscholastic Cycling Association (NICA) Mountain Biking Injury Surveillance System (ISS): Analysis of 66,588 Student Athlete-Years of Injury Data
Source: Int J Environ Res Public Health. 2021 May 29;18(11):5856. doi: 10.3390/ijerph18115856 (PMC8198101; doi:10.3390/ijerph18115856)
Supplement: Supplementary file 1 [file ijerph-18-05856-s001.zip › ijerph-1225893-supplementary.pdf]

NICA Safety Reporting Incident/Exposure Report Form

Year	◯ 2017<br>◯ 2018<br>◯ 2019<br>◯ 2020<br>◯ 2021<br>◯ 2022<br>◯ 2023<br>◯ 2024<br>◯ 2025<br>◯ 2026<br>◯ 2027
Exposure (1) or Incident (2)	__________________________________
Athlete ID	__________________________________

Thank you for participating in the NICA Student-Athlete Safety Initiative. The health and wellness of our student-athletes is NICA's highest priority. The information that you contribute to this system will help NICA better understand how to make the great sport of high school mountain biking safer.

Please remember that all of the information that you provide will always be protected to the greatest degree possible. All athlete information is stored on a highly secure, HIPAA-compliant server. NICA and its partners will never release anybody's personal health information.

For which dates are you entering your exposure?

◯ 12/09/2019-12/15/2019 ◯ 12/16/2019-12/22/2019 ◯ 12/23/2019-12/29/2019 ◯ 12/30/2019-01/05/202
0 ◯ 01/06/2020-01/12/2020 ◯ 01/13/2020-01/19/2020 ◯ 01/20/2020-01/26/2020
◯ 01/27/2020-02/02/2020 ◯ 02/03/2020-02/09/2020 ◯ 02/10/2020-02/16/2020 ◯ 02/17/2020-02/23/202
0 ◯ 02/24/2020-03/01/2020 ◯ 03/02/2020-03/08/2020 ◯ 03/09/2020-03/15/2020
◯ 03/16/2020-03/22/2020 ◯ 03/23/2020-03/29/2020 ◯ 03/30/2020-04/05/2020 ◯ 04/06/2020-04/12/202
0 ◯ 04/13/2020-04/19/2020 ◯ 04/20/2020-04/26/2020 ◯ 04/27/2020-05/03/2020
◯ 05/04/2020-05/10/2020 ◯ 05/11/2020-05/17/2020 ◯ 05/18/2020-05/24/2020 ◯ 05/25/2020-05/31/202
0 ◯ 06/01/2020-06/07/2020 ◯ 06/08/2020-06/14/2020 ◯ 06/15/2020-06/21/2020
◯ 06/22/2020-06/28/2020 ◯ 06/29/2020-07/05/2020 ◯ 07/06/2020-07/12/2020 ◯ 07/13/2020-07/19/202
0 ◯ 07/20/2020-07/26/2020 ◯ 07/27/2020-08/02/2020 ◯ 08/03/2020-08/09/2020
◯ 08/10/2020-08/16/2020 ◯ 08/17/2020-08/23/2020 ◯ 08/24/2020-08/30/2020 ◯ 08/31/2020-09/06/202
0 ◯ 09/07/2020-09/13/2020 ◯ 09/14/2020-09/20/2020 ◯ 09/21/2020-09/27/2020
◯ 09/28/2020-10/04/2020 ◯ 10/05/2020-10/11/2020 ◯ 10/12/2020-10/18/2020 ◯ 10/19/2020-10/25/202
0 ◯ 10/26/2020-11/01/2020 ◯ 11/02/2020-11/08/2020 ◯ 11/09/2020-11/15/2020
◯ 11/16/2020-11/22/2020 ◯ 11/23/2020-11/29/2020 ◯ 11/30/2020-12/06/2020 ◯ 12/07/2020-12/13/202
0 ◯ 12/14/2020-12/20/2020 ◯ 12/21/2020-12/27/2020 ◯ 12/28/2020-01/03/2021
◯ 01/04/2021-01/10/2021

In order to best understand how and why injuries are occurring, we need to have an estimate of how much the student-athletes are riding. Please provide your best estimates.

Please click the check boxes below indicating when your team had practices and races. Only include training sessions that involved biking. For each box you click, you will be asked to estimate how many student-athletes were present at each practice or ride.

	NONE	M	Tu	W	Th	F	Sa	Su
Practices	☐	☐	☐	☐	☐	☐	☐	☐
NICA-sanctioned races (note: mark "none" if no races)	☐	☐	☐	☐	☐	☐	☐	☐

IMPORTANT: YOU NEED TO CHECK ONE OF THE BOXES ABOVE!

Click the box labeled "None" if your team did not race, otherwise, choose the day that they raced.

Approximately how many student-athletes participated in Monday's practice? ________________________________

Approximately how many student-athletes participated in Tuesday's practice? ________________________________

Approximately how many student-athletes participated in Wednesday's practice? ________________________________

Approximately how many student-athletes participated in Thursday's practice? ________________________________

Approximately how many student-athletes participated in Friday's practice? ________________________________

Approximately how many student-athletes participated in Saturday's practice? ________________________________

Approximately how many student-athletes participated in Sunday's practice? ________________________________

Approximately how many student-athletes participated in Monday's race? ______________________________

Approximately how many student-athletes participated in Tuesday's race? ______________________________

Approximately how many student-athletes participated in Wednesday's race? ______________________________

Approximately how many student-athletes participated in Thursday's race? ______________________________

Approximately how many student-athletes participated in Friday's race? ______________________________

Approximately how many student-athletes participated in Saturday's race? ______________________________

Approximately how many student-athletes participated in Sunday's race? ______________________________

Number of total student-athletes practicing

____

Number of total student-athletes racing

____

Number of total athlete exposures (athletes practicing + athletes racing)

____

Number of total practices

____

Number of total races

____

Average number of student-athletes per practice

____

Average number of student-athletes per race

____

Binary code for days practiced

____
(1=Su, 2=M, 4=Tu, 8=W, 16=Th, 32=F, 64=Sa)

Binary code for days raced

____
(1=Su, 2=M, 4=Tu, 8=W, 16=Th, 32=F, 64=Sa)

Did you have any student-athlete or coach on your team who sustained reportable injuries in the past week? Click one of the options below.

◯ Yes ◯ No

Make sure you fill out an injury report for each injured person. Return to the original weekly email that you received and click on the injured rider's name to complete the injury form.

NICA Incident Report Form

You are ONLY required to fill out this form if the injury occurred at a NICA activity and meets at least one of the following criteria:

1) Warrant referral to a medical provider beyond on-site first-aid or EMS;

OR

2) Lose time from training or competition beyond day of injury;

OR

3) Miss school or work

If you do not meet these requirements, you do not need to fill this out (save your energy!). Please select 1-3 of the selections below.

☐ Warranted referral to a medical provider beyond on-site first-aid or EMS
☐ Lost time from training or competition beyond day of injury
☐ Missed school or work

League

◯ Alabama
◯ Arizona
◯ Arkansas
◯ Colorado
◯ Florida
◯ Georgia
◯ Idaho
◯ Indiana
◯ Maryland
◯ Minnesota
◯ Missouri
◯ Montana
◯ Nebraska
◯ Nevada North
◯ Nevada South
◯ New Jersey
◯ New York
◯ NorCal
◯ North Carolina
◯ Oregon
◯ Pennsylvania
◯ SoCal
◯ South Carolina
◯ Tennessee
◯ Texas
◯ Utah
◯ Virginia
◯ West Virginia
◯ Wisconsin

Team/school (enter N/A if not affiliated with a team or school) ________________________________

Team/school (enter N/A if not affiliated with a team or school) ________________________________
(alt)

Is the injured person a student-athlete, coach, volunteer, or other?

◯ student-athlete ◯ coach ◯ volunteer ◯ other

Please list the full name of the student-athlete's parent or guardian. ________________________________

Parent or guardian's email address ________________________________

Injured person's first name ________________________________

Injured person's last name ________________________________

[first_name]'s sex

◯ Male ◯ Female ◯ Other, or prefer not to identify

[first_name]'s date of birth (e.g. 3-22-2000) ________________________________

[first_name]'s race category is:

◯ Middle School
◯ Freshman
◯ Sophomore
◯ Junior Varsity (JV)
◯ Varsity
◯ 7th grade
◯ 8th grade
◯ Other or not applicable

[first_name]'s grade in school is: (e.g. 7, 8, 9, etc).

If the injured person is a coach, choose the final option.

◯ 6 ◯ 7 ◯ 8 ◯ 9 ◯ 10 ◯ 11 ◯ 12 ◯ Other or not applicable

Please enter the date and approximate time of injury
(e.g. 3-22-2015 and 11:15). ______________________________

(hidden) Today's Date - for calculation purposes ______________________________

!!!WARNING!!!

The date you entered ([date_injury]) may be incorrect, as this date/time is either too recent or in the future. Please check that it is accurate.

Where did this incident occur? Please be specific.
(e.g. Pioneer Park in Berkeley, CA). ______________________________

Which body part did [first_name] injure? These are listed in order from head to toe. Click all that apply.

☐ Concussion or possible concussion (head/brain)
☐ Head/superficial (not a concussion - scalp wound, bruise, hematoma/goose egg, etc.)
☐ Face
☐ Neck
☐ Shoulder (including collar bone)
☐ Arm (between shoulder and elbow)
☐ Elbow
☐ Forearm (between elbow and wrist)
☐ Wrist & hand
☐ Upper back (thoracic spine)
☐ Lower back (lumbar spine)
☐ Abdomen & chest
☐ Pelvis & hip
☐ Thigh (between hip and knee)
☐ Knee
☐ Leg (between knee and ankle)
☐ Foot & ankle
☐ Other

What happened in the head injury?

☐ Scalp wound
☐ Bruise
☐ Hematoma/Goose Egg
☐ Skull fracture
☐ Unknown/other

Other, please comment:

____

Did [first_name] injure the left or right foot/ankle?

◯ left ◯ right ◯ both ◯ does not apply

What was the diagnosis for the foot/ankle injury/injuries? Click all that apply.

☐ Unknown
☐ Contusion (bruise)
☐ Abrasion (scrape of the skin)
☐ Laceration (cut of the skin)
☐ Sprain (ligament injury at a joint)
☐ Strain (muscle or tendon injury)
☐ Fracture (broken bone)
☐ Dislocation (joint out of place)
☐ Unknown/other

Other, please comment:

____

Did [first_name] injure the left or right leg?

◯ left ◯ right ◯ both ◯ does not apply

What was the diagnosis for the leg injury/injuries? Click all that apply.

☐ Unknown
☐ Contusion (bruise)
☐ Abrasion (scrape of the skin)
☐ Laceration (cut of the skin)
☐ Sprain (ligament injury at a joint)
☐ Strain (muscle or tendon injury)
☐ Fracture (broken bone)
☐ Dislocation (joint out of place)
☐ Unknown/other

Other, please comment:

____

Did [first_name] injure the left or right knee?

◯ left ◯ right ◯ both ◯ does not apply

What was the diagnosis for the knee injury/injuries? Click all that apply.

☐ Unknown
☐ Contusion (bruise)
☐ Abrasion (scrape of the skin)
☐ Laceration (cut of the skin)
☐ Sprain (ligament injury at a joint)
☐ Strain (muscle or tendon injury)
☐ Fracture (broken bone)
☐ Dislocation (joint out of place)
☐ Unknown/other

Other, please comment:

____

Did [first_name] injure the left or right thigh?

◯ left ◯ right ◯ both ◯ does not apply

What was the diagnosis for the thigh injury/injuries? Click all that apply.

☐ Unknown
☐ Contusion (bruise)
☐ Abrasion (scrape of the skin)
☐ Laceration (cut of the skin)
☐ Sprain (ligament injury at a joint)
☐ Strain (muscle or tendon injury)
☐ Fracture (broken bone)
☐ Dislocation (joint out of place)
☐ Unknown/other

Other, please comment:

____

Did [first_name] injure the left or right wrist/hand?

◯ left ◯ right ◯ both ◯ does not apply

What was the diagnosis for the wrist/hand injury/injuries? Click all that apply.

☐ Unknown
☐ Contusion (bruise)
☐ Abrasion (scrape of the skin)
☐ Laceration (cut of the skin)
☐ Sprain (ligament injury at a joint)
☐ Strain (muscle or tendon injury)
☐ Fracture (broken bone)
☐ Dislocation (joint out of place)
☐ Unknown/other

Other, please comment:

____

Did [first_name] injure the left or right forearm?

◯ left ◯ right ◯ both ◯ does not apply

What was the diagnosis for the forearm injury/injuries? Click all that apply.

☐ Unknown
☐ Contusion (bruise)
☐ Abrasion (scrape of the skin)
☐ Laceration (cut of the skin)
☐ Sprain (ligament injury at a joint)
☐ Strain (muscle or tendon injury)
☐ Fracture (broken bone)
☐ Dislocation (joint out of place)
☐ Unknown/other

Other, please comment:

____

Did [first_name] injure the left or right shoulder?

◯ left ◯ right ◯ both ◯ does not apply

What was the diagnosis for the shoulder injury/injuries? Click all that apply.

☐ Unknown
☐ Contusion (bruise)
☐ Abrasion (scrape of the skin)
☐ Laceration (cut of the skin)
☐ Sprain (ligament injury at a joint)
☐ Strain (muscle or tendon injury)
☐ Fracture (broken bone)
☐ Dislocation (joint out of place)
☐ Unknown/other

Other, please comment:

____

Did [first_name] injure the left or right elbow?

◯ left ◯ right ◯ both ◯ does not apply

What was the diagnosis for the elbow injury/injuries? Click all that apply.

☐ Unknown
☐ Contusion (bruise)
☐ Abrasion (scrape of the skin)
☐ Laceration (cut of the skin)
☐ Sprain (ligament injury at a joint)
☐ Strain (muscle or tendon injury)
☐ Fracture (broken bone)
☐ Dislocation (joint out of place)
☐ Unknown/other

Other, please comment:

____

Did [first_name] injure the left or right arm?

◯ left ◯ right ◯ both ◯ does not apply

What was the diagnosis for the arm injury/injuries? Click all that apply.

☐ Unknown
☐ Contusion (bruise)
☐ Abrasion (scrape of the skin)
☐ Laceration (cut of the skin)
☐ Sprain (ligament injury at a joint)
☐ Strain (muscle or tendon injury)
☐ Fracture (broken bone)
☐ Dislocation (joint out of place)
☐ Unknown/other

Other, please comment:

____

Was [first_name] able to complete the training session or race?

◯ No ◯ Yes ◯ N/A

Was [first_name] evacuated from the trail or race course by emergency personnel?

Please note that NICA volunteers and coaches are NOT considered emergency personnel.

◯ No ◯ Yes ◯ Unknown

How was [first_name] transported?

☐ Ambulance
☐ Helicopter
☐ Private vehicle
☐ All-terrain vehicle (ATV)
☐ Other

Did the injured person go to the emergency room (ER) at any time for the injury?

This can be updated at a later point if necessary. Please include what has been done so far.

◯ No
◯ Went to ER, then sent home
◯ Went to ER, then admitted to hospital
◯ Unknown

Which health care provider(s) did [first_name] see for the injury? Click all that apply.

This can be updated at a later point if necessary. Please include what has been done so far.

☐ None
☐ Unknown
☐ Emergency medicine practitioner
☐ Urgent care clinic practitioner
☐ Athletic Trainer
☐ Physical therapist
☐ Pediatrician
☐ Family practitioner
☐ Orthopedic surgeon
☐ Sports medicine physician (non-surgical)
☐ Chiropractor
☐ Hospitalized
☐ First aid tent (NICA does not define this as a medical provider)
☐ Other

Other, please comment:

____

At which type of ride did the injury occur?

◯ Team practice (on mountain bike trails)
◯ Team practice (riding on paved roads)
◯ Race
◯ Camp
◯ Team practice (skills training)
◯ Leaders' summit
◯ NICA On-the-Bike Skills
◯ Coaches' retreat
◯ Other

Other, please comment:

____

At which point in the ride did the injury occur?

◯ Warm-up
◯ First third of the ride/race (e.g. 2nd lap of an 8 lap course)
◯ Middle third of the ride/race (e.g. 4th lap of an 8 lap course)
◯ End of the ride/race (e.g. 7th lap of an 8 lap course)
◯ Finish line
◯ Cool-down
◯ Other

Other, please comment:

____

Was the venue or route familiar to the injured rider?

◯ No, the rider had not ridden on this trail before
◯ Yes, the rider had ridden on this trail before
◯ N/A or unsure

Were any of the following associated with the injury? Click all that apply.

☐ Nothing
☐ Highly technical nature of the portion of trail (e.g. obstacle, difficult turn, loose terrain, etc.)
☐ Inexperience of the student-athlete (e.g. a more experienced rider would not have made the same mistake)
☐ Injury occurred on a turn
☐ Passing another rider or getting passed
☐ Dehydration
☐ Lack of sleep
☐ Improper nutrition
☐ Illness at the time of the ride
☐ Rider too hot
☐ Rider too cold
☐ Weather
☐ Mechanical problem (e.g. failing brakes, loose cables, inability to shift, etc.)
☐ Other

Other

Which weather condition(s) were associated with the injury?

☐ Rain
☐ Snow
☐ Ice
☐ Cold
☐ Heat
☐ Wind
☐ Hail
☐ Humidity
☐ Other

Other, please comment:

____

Please click on the type of trail condition where the injury occurred? Click all that apply.

☐ Roots
☐ Rocks
☐ Mud
☐ Snow
☐ Ice
☐ Sand
☐ Dirt
☐ Gravel
☐ Sharp turn
☐ Rut
☐ Log
☐ Water crossing
☐ Other man-made
☐ Other natural
☐ Not applicable
☐ Other

Other, please comment:

____

Please provide a description of how the incident occurred, along with any factors that may have contributed to the incident. If you have any input on how to reduce the likelihood of any similar incidents, please let us know.

Please do not use the athlete's name in this description.

(e.g. "The athlete went over his handlebars when he hit a tree root. He landed on his head and left wrist and complained of a headache. He was unable to finish the race. It would be great if we could alter this course to go around the tree or improve rider training on riding routes.")

Please provide a description or diagnosis of the injury.

Please do not use the athlete's name in this description.

(e.g. "The athlete had a concussion and broke his left wrist. He had a small abrasion to his left eye, and a bruise on his right shoulder. He was fully coherent right after the crash and walked off.")

What was the trail incline where the injury occurred?

◯ Downhill / descending
◯ Flat
◯ Uphill / ascending
◯ N/A
◯ Other

Who was leading the ride, or running the event?

(e.g. "John Smith was ride leader in charge of the ride")

____

Did the ride or event leader (that was noted in the previous question) witness the injury event?

(e.g. John Smith saw the rider crash = yes)

◯ Yes
◯ No
◯ Unsure or N/A

(OBSOLETE) What was the licensing level of the coach who was supervising the student-athlete at the time of the injury?

◯ Level 1
◯ Level 2
◯ Level 3
◯ unsure or N/A

****
If this was not a season-ending injury for [first_name], please provide the date that [first_name] was able to get back to practice or competition from the injury that occurred on [date_injury].

If this was a season-ending injury for [first_name], please consult with the student-athlete and his/her parent or guardian and estimate the date that [first_name] will be recovered enough to get back to riding outdoors.

You can complete this question at a later time when you have more information, if necessary.

____

(Hidden - not using this question anymore)********************
Was this definitely a season-ending injury for [first_name]?

Even if there was a relatively minor injury at the end of the season so the student-athlete could not complete the remainder of the season, please mark this as "yes."

◯ Yes ◯ No

Person submitting report - first name

____

Person submitting report - last name

____

Person submitting report - email address

____

Person submitting report - phone number

____

If you had any problems with this entry, please note them here.

____

(Optional) Feel free to upload any photographs that you think might be helpful in helping us to understand the injury or the trail conditions where the crash occurred.
